# A Just-in-Time Video Primer on Pneumothorax Pathophysiology and Early Management

**DOI:** 10.21980/J8891J

**Published:** 2020-04-15

**Authors:** Nicholas MacDonald, Jacob Garcia, Gregory C Kane, Xiao Chi Zhang, Dimitrios Papanagnou

**Affiliations:** * Thomas Jefferson University Hospitals, Sidney Kimmel Medical College, Philadelphia, PA; ^ Thomas Jefferson University Hospitals, Department of Medicine, Philadelphia, PA; † Thomas Jefferson University Hospitals, Department of Emergency Medicine, Philadelphia, PA

## Abstract

**Audience:**

Emergency medicine residents (interns, junior residents), medical students, and mid-level providers (physician assistants, nurse practitioners).

**Introduction:**

Pneumothorax refers to the presence of gas within the pleural space and is a relatively common clinical entity in the emergency department.^1^ Traumatic pneumothorax results from blunt or penetrating trauma to the thorax. Iatrogenic pneumothorax is a risk inherent to a number of invasive procedures and represents a significant cause of preventable morbidity.^2^ Specifically, central venous catheterization (43.8%), thoracentesis (20.1%), and barotrauma due to mechanical ventilation (9.1%) are the most frequent causes.^3^

A feared complication of pneumothorax is the development of tension pneumothorax, which involves the compression of mediastinal structures by increased pressures within the pleural space, leading to hemodynamic compromise.^4^ As tension pneumothorax is an emergent, life-threatening condition, the management of pneumothorax and the insertion of chest tubes are skills required of physicians involved in the care of injured patients, including general surgeons, intensivists, and emergency medicine physicians.^5^ The process of correcting pneumothorax is not without complication. Complications following chest tube insertion in trauma patients occur in 19% of cases,^6^ and are commonly a result of chest tubes placed by resident physicians.^7^

The authors believe that a web-based learning module addressing topics related to pneumothorax (pathophysiology, clinical manifestations, diagnosis, and management) would be beneficial to healthcare providers who are likely to encounter pneumothorax in clinical practice. Specifically, the web-based nature of the module would lend itself to convenient viewing and would allow for utilization as a just-in-time training modality. Presenting these topics in an animated format may also be a useful way of capturing the complex and three-dimensional nature of respiratory physiology. Additionally, the web-based format may be particularly appealing to digital native natives, who occupy an increasing percentage of resident physician positions.^8^ It should be noted that a number of studies have examined the use of computerized modules in medical education, and found them to be at least as useful as traditional instructional methods, and are typically associated with high rates of satisfaction among learners.^9^–^13^

**Educational Objectives:**

By the end of this module, participants should be able to:

1. Review the normal physiology of the pleural space
2. Discuss the pathophysiology of pneumothorax
3. Describe the clinical presentation of pneumothorax
4. Identify pneumothorax on a chest radiograph
5. Review treatment options for pneumothorax

**Educational Methods:**

This is a video podcast, which conveys information through animated content. It is available to learners on demand and just-in-time for practice. It may be used as a stand-alone educational tool, as a primer to other instructional methods (eg, simulation), or a just-in-time training tool.

**Research Methods:**

A small-scale study was conducted to evaluate the efficacy of this module as an educational tool. The learner group consisted of a convenience sample of 11 second-year medical students at the end of their pre-clinical training. All learners were administered the attached assessment form as a pre-test, shown the video, then asked to re-take the assessment as a post-test to assess improvement. Assessments were graded on a 10-point scale according to the attached answer key. Learners were also given the opportunity to rate the quality of the module as an educational tool, as well as to provide subjective feedback.

**Results:**

The average pre-test score across all learners was 34%. The average post-test score across all learners was 82%, representing an improvement of 48%. Learners were asked to rate their agreement with the statements, “This module effectively taught concepts related to pulmonary physiology and pneumothorax,” and, “The animated format of this module was useful for illustrating concepts related to pulmonary physiology and pneumothorax.” All learners responded with “agree” or “strongly agree” for each statement. When given the opportunity to provide subjective feedback regarding the module, learners responded with “This module is a great review! It is well organized, has effective animations, and information is clear,” and “Helpful review that explained the concepts in an accessible way!”

**Discussion:**

Results from the pre-test and post-test suggest that this module was effective in teaching concepts related to pulmonary physiology and pneumothorax. All learners reported satisfaction with the animated format in particular. These results suggest that this module would be effective as a standalone educational tool or as a primer to other instructional methods.

**Topics:**

Pneumothorax, thoracostomy, needle decompression, flipped classroom, asynchronous learning, emergency medicine.

## USER GUIDE

[Table t1-jetem-5-2-l20]

**Table t1-jetem-5-2-l20:** 

List of Resources:	
Abstract	20
User Guide	23
Appendix A: Pre/Post Test Questions	25
Appendix B: Pneumothorax Lecture Video	28
Appendix C: Pre/Post Test Answers	29

**Learner Audience:**

Medical students, Interns, Junior Residents, Mid-level providers (physician assistants, nurse practitioners)

**Time Required for Implementation:** 15 minutes

**Recommended Number of Learners per Instructor:**

This is a video podcast that can be viewed by learners on demand. It can be viewed by any number of learners, and no instructor is required. Instructors, however, have the opportunity to use this as preparatory material for a flipped classroom session or simulation.

**Topics:**

Pneumothorax, thoracostomy, needle decompression, flipped classroom, asynchronous learning, emergency medicine.

**Objectives:**

By the end of this module, participants will be able to:

1. Review the normal physiology of the pleural space
2. Discuss the pathophysiology of pneumothorax
3. Describe the clinical presentation of pneumothorax
4. Identify pneumothorax on a chest radiograph
5. Review treatment options for pneumothorax

### Linked objectives and methods

This module instructs learners on pneumothorax using an animated format, which is useful for conceptualizing the complexities of pleural physiology (objective 1), as well as the pathophysiologic mechanisms of pneumothorax (objective 2). Participants will also learn about the range of presentations of pneumothorax (objective 3), which vary based on severity of disease. In addition to animations, this module contains representative radiographic images displaying the characteristic changes seen with pneumothorax (objective 4). A discussion of the available treatment options for pneumothorax is also included, as well as animated demonstrations of these maneuvers (objective 5).

### Recommended pre-reading for instructor

Roberts and Hedges’ *Clinical Procedures in Emergency Medicine*,^14^ Section II, Chapter 10: Tube Thoracostomy.

### Results and tips for successful implementation

A small-scale study was performed to evaluate the efficacy of this module as an educational tool. The learner group consisted of a convenience sample of 11 second-year medical students at the end of their pre-clinical training. Learners were administered the attached assessment form as a pre-test, shown the module, and then asked to re-take the assessment as a post-test to assess improvement. The average pre-test score across all learners was 34%. The average post-test score across all learners was 82%, representing an improvement of 48%. Learners unanimously agreed that the module was effective for teaching concepts related to pulmonary physiology and pneumothorax. These results suggest that this module would be effective as a standalone educational tool or as a primer to other instructional methods.

### Associated content (optional)

- Pre/Post Test
- Pre/Post Test Answers
- Pneumothorax Lecture Video
  ○ https://youtu.be/UBGH6__EyqU

### Technology necessary

Computer, tablet, or mobile device

## Supplementary Information



## Figures and Tables

**Figure f1-jetem-5-2-l20:**
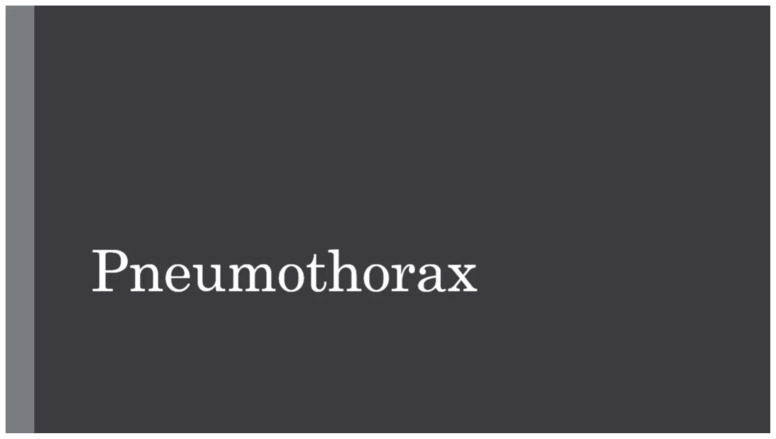
Please see associated mp4 file https://youtu.be/UBGH6__EyqU

## LEARNER MATERIALS

### Appendix A Pre/Post-Test Questions

#### MULTIPLE CHOICE QUESTIONS

1. When performing chest tube thoracostomy, which of the following describes correct positioning of the skin incision relative to the point of entry into the pleural space?
  - Inferior to the point of pleural entry
  - At the level of the point of pleural entry
  - Superior to the point of pleural entry
  - The chest tube should not enter the pleural space
2. For an unstable patient in whom tension pneumothorax is suspected, which of the following is the most appropriate first step?
  - PA and lateral chest radiographs
  - Ultrasound at the bedside
  - Supplemental oxygen and serial observations
  - Needle decompression
3. Which of the following describes the correct relationship of a rib and its corresponding neurovascular structures?
  - Neurovascular structures are located along the superior aspect of each rib
  - Neurovascular structures are located along the inferior aspect of each rib
  - Neurovascular structures are located superficial to each rib
  - There are no clinically significant neurovascular structures associated with the ribs
4. During normal tidal respiration, which of the following describes the correct relationship between pressure within the pleural space and the pressure of the surrounding atmosphere?
  - Pleural Pressure > Atmospheric Pressure
  - Pleural Pressure = Atmospheric Pressure
  - Pleural Pressure < Atmospheric Pressure
  - There are no pressures associated with the pleural space
5. A patient with a longstanding history of COPD develops sudden onset shortness of breath while working outside in his garden. Upon arrival to the ED, pneumothorax is identified on chest radiograph. Which of the following represents the appropriate classification for this patient’s pneumothorax?
  - Primary traumatic pneumothorax
  - Secondary traumatic pneumothorax
  - Primary spontaneous pneumothorax
  - Secondary spontaneous pneumothorax
6. As measured on a PA chest radiograph, which of the following represents the cutoff for the size of a spontaneous pneumothorax that would indicate the need for chest tube thoracostomy?
  - > 0.5cm
  - > 1cm
  - > 2 cm
  - > 3 cm

#### OPEN ENDED QUESTIONS

7. Describe 3 findings on PA chest radiograph that suggest the presence of tension pneumothorax.
8. Describe 5 Clinical Findings That suggest the presence of tension pneumothorax.
9. Describe the mechanism of hemodynamic collapse that can result from tension pneumothorax.
10. Using surface anatomical landmarks, describe the preferred site of needle insertion when performing a needle decompression in the treatment of pneumothorax.

### Appendix B Pneumothorax Lecture Video

[Fig f1-jetem-5-2-l20]

### Appendix C Pre/Post-Test Ansers

#### MULTIPLE CHOICE QUESTIONS

1. When performing chest tube thoracostomy, which of the following describes correct positioning of the skin incision relative to the point of entry into the pleural space?
  - **Inferior to the point of pleural entry (Refer to 10:35 in the video)**
  - At the level of the point of pleural entry
  - Superior to the point of pleural entry
  - The chest tube should not enter the pleural space
2. For an unstable patient in whom tension pneumothorax is suspected, which of the following is the most appropriate first step?
  - PA and lateral chest radiographs
  - Ultrasound at the bedside
  - Supplemental oxygen and serial observations
  - **Needle decompression (Refer to 6:37 in the video)**
3. Which of the following describes the correct relationship of a rib and its corresponding neurovascular structures?
  - Neurovascular structures are located along the superior aspect of each rib
  - **Neurovascular structures are located along the inferior aspect of each rib (Refer to 11:05 in the video)**
  - Neurovascular structures are located superficial to each rib
  - There are no clinically significant neurovascular structures associated with the ribs
4. During normal tidal respiration, which of the following describes the correct relationship between pressure within the pleural space and the pressure of the surrounding atmosphere?
  - Pleural Pressure > Atmospheric Pressure
  - Pleural Pressure = Atmospheric Pressure
  - **Pleural Pressure < Atmospheric Pressure (Refer to 0:30 in the video)**
  - There are no pressures associated with the pleural space
5. A patient with a longstanding history of COPD develops sudden onset shortness of breath while working outside in his garden. Upon arrival to the ED, pneumothorax is identified on chest radiograph. Which of the following represents the appropriate classification for this patient’s pneumothorax?
  - Primary traumatic pneumothorax
  - Secondary traumatic pneumothorax
  - Primary spontaneous pneumothorax
  - **Secondary spontaneous pneumothorax (Refer to 4:13 in the video)**
6. As measured on a PA chest radiograph, which of the following represents the cutoff for the size of a spontaneous pneumothorax that would indicate the need for chest tube thoracostomy?
  - > 0.5cm
  - > 1cm
  - > 2 cm
  - **> 3 cm (Refer to 13:30 in the video)**

#### OPEN ENDED QUESTIONS

7. Describe 3 findings on PA chest radiograph that suggest the presence of tension pneumothorax.
  **Acceptable Answers:**
  1. **Absence of lung markings extending to edge of thoracic cavity**
  2. **Flattening of diaphragm on affected side**
  3. **Increased rib spacing on affected side**
  4. **Shifting of mediastinum away from affected side.**
    **(Refer to 6:59 in the video)**
8. Describe 5 clinical findings that suggest the presence of tension pneumothorax.
  **Acceptable Answers:**
  1. **Chest pain (unilateral, pleuritic)**
  2. **Dyspnea**
  3. **Decreased breath sounds on affected side**
  4. **Hyper-resonance to percussion on affected side**
  5. **Tracheal deviation away from the pneumothorax**
  6. **Tachycardia**
  7. **Distension of neck veins**
  8. **Displacement of apex beat**
    **(Refer to 6:30 in the video)**
9. Describe the mechanism of hemodynamic collapse that can result from tension pneumothorax.
  **Answer: Increased pressure within the pleural cavity compresses mediastinal structures. At high enough pressures, this can impair venous return to the heart and ultimately result in obstructive shock. (Refer to 5:15 in the video)**
10. Using surface anatomical landmarks, describe the preferred site of needle insertion when performing a needle decompression in the treatment of pneumothorax.
  **Answer: The second intercostal space, in the mid-clavicular line. More specifically, the needle should be advanced close to the superior aspect of the 3**^**rd**^ **rib, in order to avoid the neurovascular structures along the inferior aspect of the 2**^**nd**^ **rib. This procedure may also be performed along the anterior axillary line in the 4**^**th**^ **or 5**^**th**^ **intercostal space. (Refer to 9:34 in the video)**
